# The role of plant epigenetics in biotic interactions

**DOI:** 10.1111/nph.15408

**Published:** 2018-08-29

**Authors:** Conchita Alonso, Daniela Ramos‐Cruz, Claude Becker

**Affiliations:** ^1^ Estación Biológica de Doñana Consejo Superior de Investigaciones Científicas (CSIC) Av. Américo Vespucio 26 Sevilla 41092 Spain; ^2^ Gregor Mendel Institute of Molecular Plant Biology Austrian Academy of Sciences Vienna Biocenter (VBC) Dr. Bohr Gasse 3 Vienna 1030 Austria

**Keywords:** biotic stress, DNA methylation, epigenetic, plant–animal interactions, plant–microbe interactions, priming

## Abstract

Plants are hubs of a wide range of biotic interactions with mutualist and antagonist animals, microbes and neighboring plants. Because the quality and intensity of those relationships can change over time, a fast and reversible response to stress is required. Here, we review recent studies on the role of epigenetic factors such as DNA methylation and histone modifications in modulating plant biotic interactions, and discuss the state of knowledge regarding their potential role in memory and priming. Moreover, we provide an overview of strategies to investigate the contribution of epigenetics to environmentally induced phenotypic changes in an ecological context, highlighting possible transitions from whole‐genome high‐resolution analyses in plant model organisms to informative reduced representation analyses in genomically less accessible species.

**Table:** 

Contents		
	Summary	731
I.	Biotic interactions in the context of genetic, epigenetic and environmental diversity	731
II.	Biotic interactions affect epigenetic configuration	732
III.	Plant epigenetic configuration influences biotic interactions	733
IV.	Epigenetic memory in the context of biotic interactions	734
V.	Conclusions and future research	735
	Acknowledgements	735
	Author contributions	735
	References	735

## I. Biotic interactions in the context of genetic, epigenetic and environmental diversity

Plants are a highly diversified group of sessile organisms, and as such cannot flee from changing environments. Their local persistence requires strategies that allow mitigating short‐term negative impacts without compromising future fitness (Douma *et al*., 2017). Besides numerous abiotic factors such as temperature, light, nutrient and water availability, the plants’ complex biotic environment substantially affects plant performance. While some biotic interactors are beneficial or even essential for the plant (e.g. pollinators, rhizobia, mycorrhiza), others such as herbivores, pathogens or strong competitors are detrimental. Fitness effects caused by biotic interactions vary in magnitude and impact within and among plant species and are modulated by genetic components as well as by the co‐occurrence of abiotic and biotic factors (Lucas‐Barbosa, 2016; Zust & Agrawal, 2017). For example, plant–pathogen interactions and perception of neighboring plants via light‐quality receptors influence the cross‐talk between key signaling molecules and pathways involved in defense and growth, including jasmonic acid (JA), salicylic acid (SA) and reactive oxygen species. This affects the profile of plant secondary metabolites and emitted volatile organic compounds (VOCs), which in turn has an impact on plant–herbivore and plant–pollinator interactions and, hence, on fitness (Holeski *et al*., 2012; Austin & Ballare, 2014; Lucas‐Barbosa, 2016).

**Figure 1:**
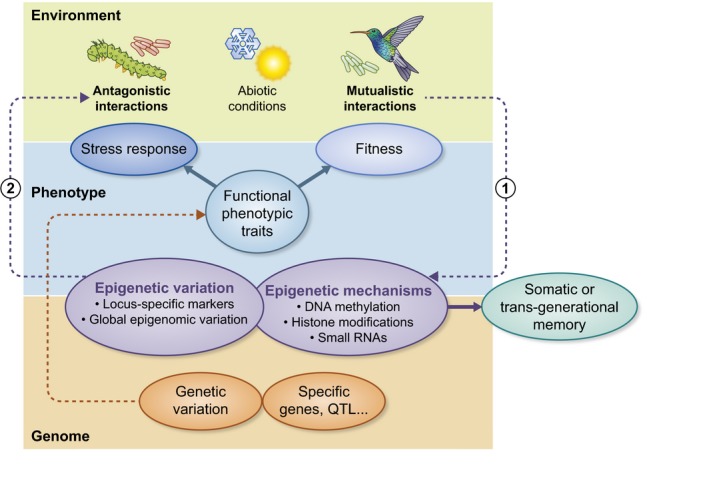
Plant phenotype is affected by abiotic conditions and diverse biotic interactions that can range from mutualistic to neutral to antagonistic. Analysis of functional phenotypic traits can help to better understand how epigenetic features contribute to plant fitness and response to biotic stress. While biotic interactions can impact the plant's epigenetic configuration (1), epigenetic features in turn influence biotic interactions (2) by modulating the plant's response. Gaining insights into functional relationships requires concurrent analysis of epigenetic variation and phenotypic trait variation between individuals exposed to contrasted biotic interactions. In addition, a better understanding of epigenetic molecular mechanisms and the epigenetic regulation of specific loci and physiological pathways is necessary to clarify epigenetic contribution to the stabilization of environmentally induced phenotypes across generations.

Besides genetic diversity and environmental components, epigenetic factors such as DNA methylation, small RNAs and post‐translational histone modifications have emerged as relevant modulators of plants’ responses to the environment (Law & Jacobsen, 2010; Lamke & Baurle, 2017) (Fig. 1). The majority of studies have focused on abiotic stress and its immediate and long‐lasting footprint on DNA methylation and histone modifications (reviewed by Kim *et al*., 2015; Pandey *et al*., 2016; Bej & Basak, 2017). By contrast, studies of biotic interactions and the links between epigenetic and phenotypic variation in that context remain sparse, probably because the scientific community still needs to define suitable strategies. Most studies have focused on the epigenetic *consequences* of biotic interactions; however, a more explicit trait‐oriented approach is required to further address the potential role of the plant epigenetic configuration in determining the quality and amplitude of those responses (Box 1). Although challenging, especially because nonmodel species are highly diverse in epigenomic features (Springer *et al*., 2016) and have limited genome information, uncovering potential associations between epigenomic configuration and phenotypic response is essential for a comprehensive understanding of evolutionary processes and for accurate predictions for crop breeding in the context of rapidly changing climate conditions (Gallusci *et al*., 2017; Richards *et al*., 2017). In the following, we will first summarize recent findings on the two‐way relationship between biotic interactions and the plant epigenome, before discussing what is and is not currently known about epigenetically regulated memory of such interactions and adaptation to them.

Box 1 **Suitable approaches to detect links between epigenetic variation and plant functional phenotypic traits.**

1

1. Direct phenotypic characterization of epigenetic mutants. Useful to characterize whole‐plant traits (i.e. individual size, architecture) and organ traits (e.g. leaf size, flower shape, fruit color) affected by loss‐of‐function of enzymes involved in DNA methylation and histone modification. Available almost exclusively for model plant species, the establishment of epigenetic recombinant inbred lines (epiRILs) in *Arabidopsis thaliana* has been particularly fruitful (see main text).
2. Somatic relationship after stress exposure. Experimental manipulation of biotic interactions (exclosure/addition of herbivores, pollinators, competitors, etc.; hand‐pollination; artificial herbivory), combined with phenotypic and epigenetic analyses of treated vs control plants. Unfortunately, similar epigenetic studies focused on abiotic stress do not usually report phenotypic analyses (Kim *et al*., 2015; but see e.g. Rendina González *et al*., 2017).
3. Transgenerational relationship after stress exposure. Experimental manipulation of biotic environment and phenotypic and epigenetic analysis of the offspring of treated vs control parents; this should also include genetic analyses. Again, epiRILs offer outstanding opportunities for this approach.
4. The use of inhibitors of DNA methyltransferases (e.g. 5‐azacytidine, zebularine) or histone deacetylases (e.g. Trichostatin A) in combination with biotic factors. Specific protocols have been successfully applied to different plant species (Alonso *et al*., 2017; Puy *et al*., 2018).
5. Ecological (or evolutionary) relationship. Phenotypic and epigenetic analysis of wild populations across environmental gradients, including substantial changes in biotic interactions (e.g. permanent exclosures, insect outbreaks). Concurrent analysis of spatial genetic and epigenetic structure would clarify their respective contribution to plant population differentiation (Herrera *et al*., 2016).

In all cases, measured phenotypic traits should include plant fitness (e.g. biomass, seed mass or number) and other traits more relevant for specific biotic interactions (e.g. leaf water content, specific leaf area, spinescence and secondary compounds for herbivory; floral pigmentation, shape and volatiles for pollination; fruit size, pigmentation and nutrient value for seed dispersal). Concurrent analyses of changes in the selection pressure imposed by biotic interactors on phenotypes of individuals with contrasted epigenetic features (1, 4) and epigenetic divergence on individuals experiencing contrasted levels of specific biotic interactions (2, 3, 5) on the same study system will certainly contribute to a more realistic understanding of the role of epigenetic variation in plant adaptation.

## II. Biotic interactions affect epigenetic configuration

Among studies of epigenetic alterations following biotic interactions, the analysis of plant–pathogen interactions prevails (Zogli & Libault, 2017). Because it would exceed the scope of the present article, we cannot discuss the plethora of plant–pathogen interactions involving noncoding RNAs, even though small RNAs have been associated with immunity in various plant species and have even recently been shown to modulate pathogen virulence in cross‐kingdom interference (Cai *et al*., 2018). Instead, we will here focus on chromatin configuration changes related to biotic interactions, and point readers interested in the role of noncoding RNAs to recent reviews on the topic (Wang *et al*., 2017; Ramirez‐Prado *et al*., 2018).

Most of what we know about the epigenetic consequences of plant pathogen attack originates from studies of the bacterial pathogen *Pseudomonas syringae* (*Pst*). In the first whole‐epigenome single‐nucleotide‐resolution analysis of a plant–microbe interaction, virulent *Pst* induced DNA methylation changes in *Arabidopsis thaliana* across all sequence contexts (CG, CHG and CHH, where H can be any base but G), whereas an avirulent strain or the defense hormone SA elicited changes only in CG and CHG methylation (Dowen *et al*., 2012). Methylation changes were frequent proximal to defense‐related genes and correlated with their transcriptional activation upon treatment, suggesting a role in the response to the pathogen. Complementary to these findings, treatment with the bacterial elicitor FLG22 resulted in a REPRESSOR OF SILENCING1 (ROS1)‐dependent demethylation of transposable elements (TEs) in proximity to defense‐related genes (Yu *et al*., 2013). Much less is known about the effects of fungal pathogens or oomycetes on the plant epigenome. Upon infection with the necrotrophic pathogen *Botrytis cinerea*,* A. thaliana* and tomato plants showed local changes in the activating histone marks H3K4me3 and H3K9ac (acetylation of lysine 9), as well as for repressive H3K27me3, although the cause–consequence relationship between epigenetic and transcriptional changes in the proximal genes remained unresolved (Crespo‐Salvador *et al*., 2018).

Epigenetic changes are not exclusively related to plant–microbe interactions. *A. thaliana* roots infected by the cyst nematode *Heterodera schachtii* showed large‐scale changes in DNA methylation and small RNA populations, with dynamic shifts across the infection stages. Although DNA methylation changes were probably over‐estimated due to lenient calling of differentially methylated regions, epigenetic changes appeared to be associated with transcriptional changes of defense‐related genes in the root (Hewezi *et al*., 2017).

In nonmodel species, for which genomic information is often lacking, changes in the patterns of DNA methylation at anonymous loci have been found to correlate with abundance of trichomes and spines (Scoville *et al*., 2011; Herrera & Bazaga, 2013), leaf palatability (Verhoeven *et al*., 2010) and long‐term differential browsing in wild‐growing individuals (Herrera & Bazaga, 2011). In *Brassica rapa*, DNA methylation changes were associated with variation in floral scent bouquet and reduced pollinator attraction induced by herbivory (Kellenberger *et al*., 2016). In future, pinpointing herbivore‐induced epigenetic changes to specific genomic loci, linking them to defensive and attractive molecular signaling networks, and investigating their potential role in priming and transgenerational transmission will require high‐resolution analyses and detailed knowledge of the genetic diversity (Holeski *et al*., 2012; Richards *et al*., 2017).

Ultimately, also plant–plant interactions mediated by plant‐derived allelochemicals or the root‐associated microbiota can leave a footprint in the chromatin configuration. For instance, breakdown products of benzoxazinoids, which are produced by many Poaceae species, upon uptake by neighbor plant roots inhibit histone deacetylase activity, resulted in hyper‐acetylation of histone lysine residues, mis‐regulation of gene expression and inhibition of root growth (Venturelli *et al*., 2015).

## III. Plant epigenetic configuration influences biotic interactions

While microbes, herbivores and neighboring plants can affect the plant's epigenome, the epigenome can in turn influence plant phenotype (e.g. Latzel *et al*., 2013), and hence can influence biotic interactions. Since the first characterization of the epigenetic basis of *Linaria vulgaris* floral mutants (Cubas *et al*., 1999) and the discovery of a defense‐promoting epiallelic locus in *A. thaliana* (Stokes *et al*., 2002), the recurring finding of epigenetic components underlying striking flower, fruit or defense phenotypes suggests a more general role of the epigenetic machinery in modulating biotic interactions in plants. In *Solanum ruiz‐lealii*, a species of hybrid origin, distinctive methylation profiles were associated with abnormal floral phenotypes, a trait with evolutionary consequences, as bumblebees do not visit plants with aberrant flowers (Marfil *et al*., 2009). In *A. thaliana,* mutants deficient in methylation maintenance were less susceptible to *Pst* (Dowen *et al*., 2012). This is in contrast to mutants deficient in the H3K36 di‐ and tri‐methyltransferase SET DOMAIN GROUP 8 (SDG8), which are more susceptible to the same pathogen because of failing epigenetic regulation of an essential defense gene upon pathogen attack (De‐La‐Pena *et al*., 2012). In rice, increased resistance to blight (*Xanthomonas oryzae*) could be induced by overexpression of a histone lysine demethylase known to target defense‐related genes (Li *et al*., 2013).

Interactions with fungal pathogens and oomycetes also seem intricately connected to the epigenetic machinery: while hypermethylated *A. thaliana* mutants were less resistant towards *Hyaloperonospora arabidopsidis* (*Hpa*) and *Fusarium oxysporum*, mutants impaired in DNA methylation establishment were less susceptible towards *Hpa* (Le *et al*., 2014; Lopez Sanchez *et al*., 2016). Defense against these pathogens also involves post‐translational histone modifications: mutants of the histone lysine methyltransferase ARABIDOPSIS TRITHORAX‐RELATED 7 (ATXR7) were more susceptible to *Hpa*, presumably because of the ATXR7‐dependent regulation of H3K4me3 at promoters of key defense genes (Xia *et al*., 2013).

Compelling evidence for the contribution of epialleles to biotic interactions comes from *A. thaliana* epigenetic recombinant inbred lines (epiRILs), originally derived from crosses of a methylation‐deficient mutant (either *met1* or *ddm1*) and the reference Col‐0 wild‐type strain (Johannes *et al*., 2009; Reinders *et al*., 2009). In the F2 generation, these isogenic lines carry mosaic epigenetic patterns that are unique for each line; epiRILs can therefore be used to identify epigenetic quantitative trait loci (epiQTLs) of associations between epigenetic and phenotypic variation. For example, epiRILs revealed considerable variation in their response to the defense‐related hormones SA and JA, often correlated for both hormone treatments (Latzel *et al*., 2012). The same authors were able to show variation among epiRILs for growth under competitive pressure by weeds and for resistance to *Pst* (Latzel *et al*., 2013), the latter being in line with the original report on pathogen resistance in *met1*‐derived epiRILs (Reinders *et al*., 2009). Together, these results suggest that epigenetic variation might contribute to variation in plant phenotypic traits observed also in natural populations. Very often, this epigenetic variation resides at transposable elements (TEs) or TE residuals. Environment‐induced epigenetic changes at these TE loci can influence gene expression and thus mediate phenotypic variation (Dubin *et al*., 2018). Studies into the environmental responsiveness of TEs have so far dealt exclusively with abiotic stress, and the role of TE‐mediated epigenetic regulation during biotic interactions awaits investigation.

## IV. Epigenetic memory in the context of biotic interactions

A continuously debated question is whether environment‐induced epigenetic effects, including those arising from biotic interactions, play a role in memory and acclimation to changing environments. In this context, one has to differentiate between somatic memory (within the life cycle of a plant), parental or intergenerational effects (in the direct offspring), and true transgenerational effects (stable for at least two generations) (Lamke & Baurle, 2017). Although priming and somatic memory to pathogens has been repeatedly reported, only very few studies have investigated the epigenetic contribution to this phenomenon (reviewed by Crisp *et al*., 2016; Lamke & Baurle, 2017). Eliciting a defense response using acibenzolar *S*‐methyl (BTH) caused changes in H3K4me2 and H3K4me3 as well as in H3 and H4 acetylation in several promoters of WRKY transcription factors; these chromatin changes in turn primed the plant for a subsequent water infiltration stress (Jaskiewicz *et al*., 2011). A recent study has highlighted a molecular player in the inverse scenario, the prevention of priming: in *A. thaliana*, the histone chaperone CHROMATIN ASSEMBLY FACTOR 1 (CAF1) prevents the establishment of a primed defense state and a loss of plant vigor by regulating nucleosome occupancy and deposition of H3K4me3 at transcription start sites of defense response genes (Mozgova *et al*., 2015).

In the context of inter‐ or transgenerational memory, the offspring of *A. thaliana* plants that had been exposed to *Pst* showed increased resistance to *Hpa*, indicating a broad‐spectrum transgenerational defence priming. Interestingly, this phenotype was mimicked by the *drm1drm2cmt3* (*ddc*) mutant impaired in RNA‐directed DNA methylation (RdDM), implicating DNA methylation in memory establishment (Luna *et al*., 2012). The priming to *Pst*/*Hpa* as well as intergenerational memory of herbivory by caterpillars in *A. thaliana* and tomato (Rasmann *et al*., 2012) persisted only for a single stress‐free generation, indicating transcriptional plasticity in response to stress and the presence of a tightly regulated and robust resetting mechanism to prevent chromatin changes from being stably inherited.

In contrast to mammals, no major resetting events of the epigenetic landscape have been observed in the plant germline (Kawashima & Berger, 2014). However, in *A. thaliana*, hyperosmosis‐induced stress priming was found to be erased in the male germ line dependent upon the DNA glycosylase DEMETER (Wibowo *et al*., 2016). This is in line with recent findings that vegetative propagation of plants, either via cell culture or somatic embryogenesis, leads to severely altered epigenetic states, indicating that accurate reconfiguration of the epigenome occurs in the process of sexual reproduction (Stroud *et al*., 2013; Han *et al*., 2018; Wibowo *et al*., 2018). Finally, two elegant forward genetics screens in *A. thaliana* for suppressors of transgenerational epigenetic inheritance of heat‐ and cold‐induced effects, respectively, uncovered major components of the DNA methylation machinery (DDM1, MOM1) and the H3K27me3 demethylase ELF6 (Crevillen *et al*., 2014; Iwasaki & Paszkowski, 2014).

## V. Conclusions and future research

Research into the relationship between epigenetics and biotic interactions has major potential to deliver answers to urgent questions regarding rapid plant adaptation, phenotypic plasticity and crop improvement. Contrary to abiotic stress treatments, biotic interactions are highly context‐dependent and, thus, more difficult to standardize, which makes comparisons of experiments across species and laboratories more challenging. A more explicit trait‐oriented approach (Box 1), similar to what had been proposed in the past for the study of interferences in the selective roles of multiple biotic interactions (Strauss *et al*., 2005), and concurrent analyses of spatial genetic and epigenetic structure (Herrera *et al*., 2016), could be instrumental to understanding the epigenetic component behind plant responses to complex natural environments.

Because complex biotic interactions must be studied in an ecological context, genetic and genomic information on the species involved is often sparse. In future, molecular biologists, genomicists and ecologists should join forces to place the molecular mechanisms involved in such interactions (e.g. induced plant defenses) in an ecological perspective. In the process, interdisciplinary research can make use of the recent technological leaps in genome sequencing and assembly to develop genomic tools and resources suitable to analyze epigenetic responses in nonmodel organisms. Moving away from the methylation analysis at anonymous markers, widely used in ecology, has been more challenging than expected, due to both technological limitations and costs (Schrey *et al*., 2013). At this point, there are several paths to move forward, always having in mind that concurrent analysis of genetic variation in the studied populations is indispensable to disentangle epigenetic from genetic effects. The first route is to study ‘classical’ or more recently established model species and their close relatives in the field (e.g. Liston *et al*., 2014; Kawakatsu *et al*., 2016). While this has the disadvantage of limiting the ecological questions that can be addressed, it offers a realistic chance of understanding epigenetic regulation of specific biotic interactions at a mechanistic level. The second option is the semi‐informed analysis of a large random set of epigenomic markers, making use of the (further) development of novel tools for analysing epigenetic markers in nonreference genomes (e.g. van Gurp *et al*., 2016; Trucchi *et al*., 2016). Although detailed functional analyses will probably not be possible using these approaches, they hold the potential to deliver a whole‐genome view of environment‐dependent epigenetic patterns and to assess epigenome–environment correlations with statistical rigor and in large populations. The third path involves the identification – in a limited number of species and using high‐resolution analyses – of key loci associated with a particular biotic interaction. After establishing that these loci undergo epigenetic changes in response to biotic stress, subsequent analyses can be limited to these loci, e.g. using target enrichment strategies, allowing for large sample numbers and analysis of correlation with phenotypic analyses. Two recent studies on epigenetic associations with glucosinolate production illustrated the potential of such an approach (Xue *et al*., 2015; Aller *et al*., 2018). Ideally, all of these approaches can be applied in species with contrasting life histories (e.g. annual vs perennial; sexual vs asexual) and/or ecological features. Progress along the above pathways can extend ecological epigenetics to studying the full spectrum of plant–animal, plant–microbe and plant–plant interaction scenarios, to contribute to a more comprehensive understanding of how plants will deal with a changing environment.

## Author contributions

C.A. and C.B. defined the scope; C.A., D.R‐C. and C.B. contributed to literature analysis and writing.

## References

[nph15408-bib-0001] Aller EST , Jagd LM , Kliebenstein DJ , Burow M . 2018 Comparison of the relative potential for epigenetic and genetic variation to contribute to trait stability. G3 (Bethesda) 8: 1733–1746. 29563187 10.1534/g3.118.200127 PMC5940164

[nph15408-bib-0002] Alonso C , Medrano M , Pérez R , Bazaga P , Herrera CM . 2017 Tissue‐specific response to experimental demethylation at seed germination in the non‐model herb *Erodium cicutarium* . Epigenomes 1: 16.

[nph15408-bib-0003] Austin AT , Ballare CL . 2014 Plant interactions with other organisms: molecules, ecology and evolution. New Phytologist 204: 257–260. 25236166 10.1111/nph.13062

[nph15408-bib-0004] Bej S , Basak J . 2017 Abiotic stress induced epigenetic modifications in plants: how much do we know? In: Rajewsky N, Jurga S, Barciszewski J, eds. Plant epigenetics. Cham, Switzerland: Springer, 493–512.

[nph15408-bib-0005] Cai Q , Qiao L , Wang M , He B , Lin FM , Palmquist J , Huang SD , Jin H . 2018 Plants send small RNAs in extracellular vesicles to fungal pathogen to silence virulence genes. Science 360: 1126–1129. 29773668 10.1126/science.aar4142 PMC6442475

[nph15408-bib-0006] Crespo‐Salvador O , Escamilla‐Aguilar M , Lopez‐Cruz J , Lopez‐Rodas G , Gonzalez‐Bosch C . 2018 Determination of histone epigenetic marks in *Arabidopsis* and tomato genes in the early response to *Botrytis cinerea* . Plant Cell Reports 37: 153–166. 29119291 10.1007/s00299-017-2218-9

[nph15408-bib-0007] Crevillen P , Yang H , Cui X , Greeff C , Trick M , Qiu Q , Cao X , Dean C . 2014 Epigenetic reprogramming that prevents transgenerational inheritance of the vernalized state. Nature 515: 587–590. 25219852 10.1038/nature13722 PMC4247276

[nph15408-bib-0008] Crisp PA , Ganguly D , Eichten SR , Borevitz JO , Pogson BJ . 2016 Reconsidering plant memory: intersections between stress recovery, RNA turnover, and epigenetics. Science Advances 2: e1501340. 26989783 10.1126/sciadv.1501340 PMC4788475

[nph15408-bib-0009] Cubas P , Vincent C , Coen E . 1999 An epigenetic mutation responsible for natural variation in floral symmetry. Nature 401: 157–161. 10490023 10.1038/43657

[nph15408-bib-0010] De‐La‐Pena C , Rangel‐Cano A , Alvarez‐Venegas R . 2012 Regulation of disease‐responsive genes mediated by epigenetic factors: interaction of *Arabidopsis–Pseudomonas* . Molecular Plant Pathology 13: 388–398. 22023111 10.1111/j.1364-3703.2011.00757.x PMC6638851

[nph15408-bib-0501] Douma JC , Vermeulen PJ , Poelman EH , Dicke M , Anten NPR . 2017 When does it pay off to prime for defense? A modeling analysis. New Phytologist 216: 782–797. 28892162 10.1111/nph.14771 PMC5659137

[nph15408-bib-0011] Dowen RH , Pelizzola M , Schmitz RJ , Lister R , Dowen JM , Nery JR , Dixon JE , Ecker JR . 2012 Widespread dynamic DNA methylation in response to biotic stress. Proceedings of the National Academy of Sciences, USA 109: E2183–E2191. 10.1073/pnas.1209329109 PMC3420206 22733782

[nph15408-bib-0012] Dubin MJ , Mittelsten Scheid O , Becker C . 2018 Transposons: a blessing curse. Current Opinion in Plant Biology 42: 23–29. 29453028 10.1016/j.pbi.2018.01.003

[nph15408-bib-0013] Gallusci P , Dai Z , Genard M , Gauffretau A , Leblanc‐Fournier N , Richard‐Molard C , Vile D , Brunel‐Muguet S . 2017 Epigenetics for plant improvement: current knowledge and modeling avenues. Trends in Plant Science 22: 610–623. 28587758 10.1016/j.tplants.2017.04.009

[nph15408-bib-0014] van Gurp TP , Wagemaker NC , Wouters B , Vergeer P , Ouborg JN , Verhoeven KJ . 2016 epiGBS: reference‐free reduced representation bisulfite sequencing. Nature Methods 13: 322–324. 26855363 10.1038/nmeth.3763

[nph15408-bib-0015] Han Z , Crisp PA , Stelpflug S , Kaeppler S , Li Q , Springer NM . 2018 Targeted epigenomic changes to the maize methylome resulting from tissue culture. *bioRxiv*: 242081. 10.1534/genetics.118.300987 PMC6063226 29848487

[nph15408-bib-0016] Herrera CM , Bazaga P . 2011 Untangling individual variation in natural populations: ecological, genetic and epigenetic correlates of long‐term inequality in herbivory. Molecular Ecology 20: 1675–1688. 21466603 10.1111/j.1365-294X.2011.05026.x

[nph15408-bib-0017] Herrera CM , Bazaga P . 2013 Epigenetic correlates of plant phenotypic plasticity: DNA methylation differs between prickly and nonprickly leaves in heterophyllous *Ilex aquifolium* (Aquifoliaceae) trees. Botanical Journal of the Linnean Society 171: 441–452.

[nph15408-bib-0018] Herrera CM , Medrano M , Bazaga P . 2016 Comparative spatial genetics and epigenetics of plant populations: heuristic value and a proof of concept. Molecular Ecology 25: 1653–1664. 26850938 10.1111/mec.13576

[nph15408-bib-0019] Hewezi T , Lane T , Piya S , Rambani A , Rice JH , Staton M . 2017 Cyst nematode parasitism induces dynamic changes in the root epigenome. Plant Physiology 174: 405–420. 28298479 10.1104/pp.16.01948 PMC5411145

[nph15408-bib-0020] Holeski LM , Jander G , Agrawal AA . 2012 Transgenerational defense induction and epigenetic inheritance in plants. Trends in Ecology & Evolution 27: 618–626. 22940222 10.1016/j.tree.2012.07.011

[nph15408-bib-0021] Iwasaki M , Paszkowski J . 2014 Identification of genes preventing transgenerational transmission of stress‐induced epigenetic states. Proceedings of the National Academy of Sciences, USA 111: 8547–8552. 10.1073/pnas.1402275111 PMC4060648 24912148

[nph15408-bib-0022] Jaskiewicz M , Conrath U , Peterhansel C . 2011 Chromatin modification acts as a memory for systemic acquired resistance in the plant stress response. EMBO Reports 12: 50–55. 21132017 10.1038/embor.2010.186 PMC3024125

[nph15408-bib-0023] Johannes F , Porcher E , Teixeira FK , Saliba‐Colombani V , Simon M , Agier N , Bulski A , Albuisson J , Heredia F , Audigier P *et al* 2009 Assessing the impact of transgenerational epigenetic variation on complex traits. PLoS Genetics 5: e1000530. 19557164 10.1371/journal.pgen.1000530 PMC2696037

[nph15408-bib-0024] Kawakatsu T , Huang SC , Jupe F , Sasaki E , Schmitz RJ , Urich MA , Castanon R , Nery JR , Barragan C , He Y *et al* 2016 Epigenomic diversity in a global collection of *Arabidopsis thaliana* accessions. Cell 166: 492–505. 27419873 10.1016/j.cell.2016.06.044 PMC5172462

[nph15408-bib-0025] Kawashima T , Berger F . 2014 Epigenetic reprogramming in plant sexual reproduction. Nature Reviews Genetics 15: 613–624. 10.1038/nrg3685 25048170

[nph15408-bib-0026] Kellenberger RT , Schluter PM , Schiestl FP . 2016 Herbivore‐induced DNA demethylation changes floral signalling and attractiveness to pollinators in *Brassica rapa* . PLoS ONE 11: e0166646. 27870873 10.1371/journal.pone.0166646 PMC5117703

[nph15408-bib-0027] Kim JM , Sasaki T , Ueda M , Sako K , Seki M . 2015 Chromatin changes in response to drought, salinity, heat, and cold stresses in plants. Frontiers in Plant Science 6: 114. 25784920 10.3389/fpls.2015.00114 PMC4345800

[nph15408-bib-0028] Lamke J , Baurle I . 2017 Epigenetic and chromatin‐based mechanisms in environmental stress adaptation and stress memory in plants. Genome Biology 18: 124. 28655328 10.1186/s13059-017-1263-6 PMC5488299

[nph15408-bib-0029] Latzel V , Allan E , Bortolini Silveira A , Colot V , Fischer M , Bossdorf O . 2013 Epigenetic diversity increases the productivity and stability of plant populations. Nature Communications 4: 2875. 10.1038/ncomms3875 24285012

[nph15408-bib-0030] Latzel V , Zhang Y , Karlsson Moritz K , Fischer M , Bossdorf O . 2012 Epigenetic variation in plant responses to defence hormones. Annals of Botany 110: 1423–1428. 22543179 10.1093/aob/mcs088 PMC3489142

[nph15408-bib-0031] Law JA , Jacobsen SE . 2010 Establishing, maintaining and modifying DNA methylation patterns in plants and animals. Nature Reviews Genetics 11: 204–220. 10.1038/nrg2719 PMC3034103 20142834

[nph15408-bib-0032] Le TN , Schumann U , Smith NA , Tiwari S , Au PC , Zhu QH , Taylor JM , Kazan K , Llewellyn DJ , Zhang R *et al* 2014 DNA demethylases target promoter transposable elements to positively regulate stress responsive genes in *Arabidopsis* . Genome Biology 15: 458. 25228471 10.1186/s13059-014-0458-3 PMC4189188

[nph15408-bib-0033] Li T , Chen X , Zhong X , Zhao Y , Liu X , Zhou S , Cheng S , Zhou DX . 2013 Jumonji C domain protein JMJ705‐mediated removal of histone H3 lysine 27 trimethylation is involved in defense‐related gene activation in rice. Plant Cell 25: 4725–4736. 24280387 10.1105/tpc.113.118802 PMC3875746

[nph15408-bib-0034] Liston A , Cronn R , Ashman TL . 2014 *Fragaria*: a genus with deep historical roots and ripe for evolutionary and ecological insights. American Journal of Botany 101: 1686–1699. 25326614 10.3732/ajb.1400140

[nph15408-bib-0035] Lopez Sanchez A , Stassen JH , Furci L , Smith LM , Ton J . 2016 The role of DNA (de)methylation in immune responsiveness of *Arabidopsis* . Plant Journal 88: 361–374. 27341062 10.1111/tpj.13252 PMC5132069

[nph15408-bib-0036] Lucas‐Barbosa D . 2016 Integrating studies on plant–pollinator and plant–herbivore interactions. Trends in Plant Science 21: 125–133. 26598297 10.1016/j.tplants.2015.10.013

[nph15408-bib-0037] Luna E , Bruce TJ , Roberts MR , Flors V , Ton J . 2012 Next‐generation systemic acquired resistance. Plant Physiology 158: 844–853. 22147520 10.1104/pp.111.187468 PMC3271772

[nph15408-bib-0038] Marfil CF , Camadro EL , Masuelli RW . 2009 Phenotypic instability and epigenetic variability in a diploid potato of hybrid origin, *Solanum ruiz‐lealii* . BMC Plant Biology 9: 21. 19232108 10.1186/1471-2229-9-21 PMC2656509

[nph15408-bib-0039] Mozgova I , Wildhaber T , Liu Q , Abou‐Mansour E , L'Haridon F , Metraux JP , Gruissem W , Hofius D , Hennig L . 2015 Chromatin assembly factor CAF‐1 represses priming of plant defence response genes. Nature Plants 1: 15127. 27250680 10.1038/nplants.2015.127

[nph15408-bib-0040] Pandey G , Sharma N , Sahu PP , Prasad M . 2016 Chromatin‐based epigenetic regulation of plant abiotic stress response. Current Genomics 17: 490–498. 28217005 10.2174/1389202917666160520103914 PMC5282600

[nph15408-bib-0041] Puy J , Dvořáková H , Carmona CP , de Bello F , Hiiesalu I , Latzel V . 2018 Improved demethylation in ecological epigenetic experiments: testing a simple and harmless foliar demethylation application. Methods in Ecology and Evolution 9: 744–753.

[nph15408-bib-0042] Ramirez‐Prado JS , Abulfaraj AA , Rayapuram N , Benhamed M , Hirt H . 2018 Plant immunity: from signaling to epigenetic control of defense. Trends in Plant Science. doi: 10.1016/j.tplants.2018.06.004. 29970339

[nph15408-bib-0043] Rasmann S , De Vos M , Casteel CL , Tian D , Halitschke R , Sun JY , Agrawal AA , Felton GW , Jander G . 2012 Herbivory in the previous generation primes plants for enhanced insect resistance. Plant Physiology 158: 854–863. 22209873 10.1104/pp.111.187831 PMC3271773

[nph15408-bib-0044] Reinders J , Wulff BB , Mirouze M , Mari‐Ordonez A , Dapp M , Rozhon W , Bucher E , Theiler G , Paszkowski J . 2009 Compromised stability of DNA methylation and transposon immobilization in mosaic *Arabidopsis* epigenomes. Genes & Development 23: 939–950. 19390088 10.1101/gad.524609 PMC2675864

[nph15408-bib-0502] Rendina González AP , Dumalasová V , Rosenthal J , Skuhrovec J , Latzel V . 2017 The role of transgenerational effects in adaptation of clonal offspring of white clover (*Trifolium repens*) to drought and herbivory. Evolutionary Ecology 31: 345–361.

[nph15408-bib-0045] Richards CL , Alonso C , Becker C , Bossdorf O , Bucher E , Colome‐Tatche M , Durka W , Engelhardt J , Gaspar B , Gogol‐Doring A *et al* 2017 Ecological plant epigenetics: evidence from model and non‐model species, and the way forward. Ecology Letters 20: 1576–1590. 29027325 10.1111/ele.12858

[nph15408-bib-0046] Schrey AW , Alvarez M , Foust CM , Kilvitis HJ , Lee JD , Liebl AL , Martin LB , Richards CL , Robertson M . 2013 Ecological epigenetics: beyond MS‐AFLP. Integrative and Comparative Biology 53: 340–350. 23583961 10.1093/icb/ict012

[nph15408-bib-0047] Scoville AG , Barnett LL , Bodbyl‐Roels S , Kelly JK , Hileman LC . 2011 Differential regulation of a MYB transcription factor is correlated with transgenerational epigenetic inheritance of trichome density in *Mimulus guttatus* . New Phytologist 191: 251–263. 21352232 10.1111/j.1469-8137.2011.03656.x PMC3107365

[nph15408-bib-0048] Springer NM , Lisch D , Li Q . 2016 Creating order from chaos: epigenome dynamics in plants with complex genomes. Plant Cell 28: 314–325. 26869701 10.1105/tpc.15.00911 PMC4790878

[nph15408-bib-0049] Stokes TL , Kunkel BN , Richards EJ . 2002 Epigenetic variation in *Arabidopsis* disease resistance. Genes & Development 16: 171–182. 11799061 10.1101/gad.952102 PMC155322

[nph15408-bib-0050] Strauss SY , Sahli H , Conner JK . 2005 Toward a more trait‐centered approach to diffuse (co)evolution. New Phytologist 165: 81–89. 15720623 10.1111/j.1469-8137.2004.01228.x

[nph15408-bib-0051] Stroud H , Ding B , Simon SA , Feng S , Bellizzi M , Pellegrini M , Wang GL , Meyers BC , Jacobsen SE . 2013 Plants regenerated from tissue culture contain stable epigenome changes in rice. eLife 2: e00354. 23539454 10.7554/eLife.00354 PMC3601819

[nph15408-bib-0052] Trucchi E , Mazzarella AB , Gilfillan GD , Lorenzo MT , Schonswetter P , Paun O . 2016 BsRADseq: screening DNA methylation in natural populations of non‐model species. Molecular Ecology 25: 1697–1713. 26818626 10.1111/mec.13550 PMC4949719

[nph15408-bib-0053] Venturelli S , Belz RG , Kamper A , Berger A , von Horn K , Wegner A , Bocker A , Zabulon G , Langenecker T , Kohlbacher O *et al* 2015 Plants release precursors of histone deacetylase inhibitors to suppress growth of competitors. Plant Cell 27: 3175–3189. 26530086 10.1105/tpc.15.00585 PMC4682303

[nph15408-bib-0054] Verhoeven KJ , Jansen JJ , van Dijk PJ , Biere A . 2010 Stress‐induced DNA methylation changes and their heritability in asexual dandelions. New Phytologist 185: 1108–1118. 20003072 10.1111/j.1469-8137.2009.03121.x

[nph15408-bib-0055] Wang J , Meng X , Dobrovolskaya OB , Orlov YL , Chen M . 2017 Non‐coding RNAs and their roles in stress response in plants. Genomics, Proteomics & Bioinformatics 15: 301–312. 10.1016/j.gpb.2017.01.007 PMC5673675 29017967

[nph15408-bib-0056] Wibowo A , Becker C , Durr J , Price J , Staepen S , Hilton S , Putra H , Papareddy R , Saintain Q , Harvey S *et al* 2018 Incomplete reprogramming of cell‐specific epigenetic marks during asexual reproduction leads to heritable phenotypic variation in plants. *bioRxiv*: 267955. 10.1073/pnas.1805371115 PMC6166847 30201727

[nph15408-bib-0057] Wibowo A , Becker C , Marconi G , Durr J , Price J , Hagmann J , Papareddy R , Putra H , Kageyama J , Becker J *et al* 2016 Hyperosmotic stress memory in *Arabidopsis* is mediated by distinct epigenetically labile sites in the genome and is restricted in the male germline by DNA glycosylase activity. eLife 5: e13546. 27242129 10.7554/eLife.13546 PMC4887212

[nph15408-bib-0058] Xia S , Cheng YT , Huang S , Win J , Soards A , Jinn TL , Jones JD , Kamoun S , Chen S , Zhang Y *et al* 2013 Regulation of transcription of nucleotide‐binding leucine‐rich repeat‐encoding genes SNC1 and RPP4 via H3K4 trimethylation. Plant Physiology 162: 1694–1705. 23690534 10.1104/pp.113.214551 PMC3707539

[nph15408-bib-0059] Xue M , Long J , Jiang Q , Wang M , Chen S , Pang Q , He Y . 2015 Distinct patterns of the histone marks associated with recruitment of the methionine chain‐elongation pathway from leucine biosynthesis. Journal of Experimental Botany 66: 805–812. 25428994 10.1093/jxb/eru440 PMC4321544

[nph15408-bib-0060] Yu A , Lepere G , Jay F , Wang J , Bapaume L , Wang Y , Abraham AL , Penterman J , Fischer RL , Voinnet O *et al* 2013 Dynamics and biological relevance of DNA demethylation in Arabidopsis antibacterial defense. Proceedings of the National Academy of Sciences, USA 110: 2389–2394. 10.1073/pnas.1211757110 PMC3568381 23335630

[nph15408-bib-0061] Zogli P , Libault M . 2017 Plant response to biotic stress: is there a common epigenetic response during plant‐pathogenic and symbiotic interactions? Plant Science 263: 89–93. 28818387 10.1016/j.plantsci.2017.07.008

[nph15408-bib-0062] Zust T , Agrawal AA . 2017 Trade‐offs between plant growth and defense against insect herbivory: an emerging mechanistic synthesis. Annual Review of Plant Biology 68: 513–534. 10.1146/annurev-arplant-042916-040856 28142282

